# Denoising of blasting vibration signals based on CEEMDAN-ICA algorithm

**DOI:** 10.1038/s41598-023-47755-9

**Published:** 2023-11-27

**Authors:** Bai Wenjun, Chang Yingjie

**Affiliations:** Shaanxi Provincial Land Engineering Construction Group Northwest Branch, Yulin, 714000 Shaanxi China

**Keywords:** Civil engineering, Techniques and instrumentation

## Abstract

Monitoring of blasting vibration signals can make the collected blasting signals noisy due to various factors such as on-site actual construction conditions, equipment, and instruments. Thus, the acquired signals should be preprocessed before analyzing the blasting vibration signals. The current study proposes a blasting vibration denoising method based on CEEMDAN-ICA to alleviate the noise component in the blasting signals effectively. The collected signal is first decomposed through the CEMMDAN algorithm to extract the IMF components of different frequency bands. Next, the collected signal is estimated using the ICA algorithm to attain corresponding ICA components. Finally, the arrangement entropy of the ICA components is calculated for signal reconstruction to attain a small noise blasting vibration signal. Simulations are performed to evaluate the feasibility of the presented algorithm and compare its efficiency with the traditional algorithms. The results demonstrate that this algorithm has specific advantages over other algorithms, which can more accurately denoise the original signal and retain the effective signals, providing a new denoising method for subsequent signal analysis.

## Introduction

The blasting vibration signal analysis is the primary research approach of railway, highway engineering, and other engineering circles to verify the blasting vibration effect, which has an adverse non-negligible impact on adjacent structures. However, in the actual construction site, the collected signal waveform will deviate from the baseline and the zero-drift phenomenon due to the complex environment of the construction site and the influence of signal sampling instruments^1^. Therefore, the signal has a trend term, which makes a significant error in the blasting vibration signal analysis, making the obtained results inconsistent with the actual situation^2–4^.

Therefore, the noise and trend signals should be processed before analyzing the signal. Currently, the most commonly used signal denoising methods include wavelet threshold, wavelet packet threshold denoising^5–7^, empirical mode decomposition (EMD)^8^, Hilbert Huang (HHT) algorithm^9^, and other approaches. Among them, wavelet and wavelet packet denoising methods were utilized more early. Luo et al.^10^ studied the application of an improved wavelet algorithm based on the autoregressive power spectrum in signal denoising; Huang et al.^11^ analyzed the collected vibration signals of mountain tunnel blasting using the wavelet algorithm. Later, with the deepening of research, the selection of wavelet basis functions determines the researchers' wavelet and wavelet packet threshold results, leading to varying degrees of analysis results with significant randomness. Zhou et al.^12^ utilized EMD and wavelet joint algorithms to improve the purity of blasting vibration signals. Qiao et al.^13^ proposed multi-scale eigenvalue EMD algorithm (ME-EMD), and carried out noise reduction for geomagnetic signals, effectively improving the accuracy of signals. However, EMD algorithms may cause signal distortion due to the low signal-to-noise ratio (SNR), which can lead to endpoint effects and modal aliasing.

Therefore, to overcome the deficiencies of EMD methods, scientists have further proposed the ensemble empirical mode decomposition (EEMD) method and the complete ensemble empirical mode decomposition with adaptive noise (CEEMDAN) approach based on the EMD algorithm. The above two methods are based on the EMD approach and incorporate the Gaussian white noise into the original signal to eliminate the signal mode aliasing phenomenon. Compared with the EEMD method, the CEEMDAN method adds a pair of auxiliary white noise with opposite numbers to each other to make the processing of the results more accurate. For example, Peng et al.^14^ proposed a CEEMDAN smooth noise reduction model for underwater blasting vibration signals. Ma et al.^15^ proposed a joint CEEMDAN-MPE-HT algorithm to verify the blasting vibration signals’ time–frequency features. Hu et al.^16^ presented a vibration signal denoising approach through the CEEMDAN algorithm and applied it to practical engineering. Yang et al.^17^ combined CEEMDAN and wavelet packet threshold processing method for signal processing analysis, and compared with the traditional algorithm, verified the superiority of the combined algorithm.

When the prior signal is unknown, independent component analysis (ICA)^18^can self-adaptively decompose the independent components and further process the noise signal in the signal. ICA is extensively utilized in image processing, signal processing, and other related areas^19–21^.

In order to effectively reduce the influence of noise in blasting vibration signals on subsequent analysis, this paper proposes a blasting vibration denoising approach by combining the CEEMDAN and ICA algorithms. Simulation experiments verify the combined approach’s feasibility, and noise reduction processing is performed on the measured blasting vibration signals. This method can overcome the EMD mode aliasing phenomenon and provide strong robustness for noise reduction.

## Basic principle

### Decomposition concept of the CEEMDAN algorithm

As the enhanced version of the EEMD algorithm, the CEEMDAN algorithm is presented to solve the modal aliasing phenomenon of the EMD approach. In the EEMD method, the Gaussian white noise signal is added to the collected signal many times before the EMD decomposition, and the average IMF component obtained by decomposition is calculated to obtain the final component. The CEEMDAN algorithm is similar to the EEMD method because it adds two mutually opposite Gaussian white noises to the collected signal and performs the EMD decomposition. Compared to the EEMD and EMD methods, the CEEMDAN method’s reconstruction error is almost zero, and it can also solve the problem of high calculational cost.

The CEEMDAN algorithm includes the following steps:

1. First, a Gaussian white noise $n_{i} (t)$ with a normal distribution is added to the original signal $s(t)$. The nth blasting vibration signal is described as follows:
  1 $$ s_{i} (t) = s(t) + n_{i} (t) $$
  where *i* is the number of times to add normal Gaussian white noise $i = 1,2,...,I$. The first-order IMF_*1*_ component is calculated, and the EMD decomposition is accomplished on the $s_{i} (t)$ added with white noise for the *i*th time to attain the IMF_*i1*_ component added with white noise,
  2 $$ IMF_{1} = \frac{1}{I}\sum\limits_{i = 1}^{I} {IMF_{i1} } $$

The residual component after removing the first-order component *IMF*_*1*_ is:

3 $$ r_{1} (t) = s(t) - IMF_{1} $$

2. The *j*-th modal function component obtained from the EMD decomposition of the signal to be processed is defined as $E_{J} ( \cdot )$, and the Gaussian white noise $n_{i} (t)$ is added to the residual component $r_{1} (t)$. The EMD method is utilized for decomposing the signal $r_{1} (t) + n_{i} (t)$ again, and the IMF2 component is obtained as follows:
  4 $$ IMF_{2} = \frac{1}{I}\sum\limits_{i = 1}^{I} {E\left[ {r_{1} (t) + n_{1} (t)} \right]} $$

The residual component after removing the second-order component *IMF*_2_ is:

5 $$ r_{2} (t) = r_{1} (t) - IMF_{2} $$

3. When $i \ge 3$ is selected, the residual component after removing the $IMF_{i}$ component is:
  6 $$ r_{i} (t) = r_{i - 1} (t) - IMF_{i} $$
4. The first IMF component of signal $r_{i} (t) + n_{i} (t)$ is extracted, and the $i + 1$-th modal function components are defined as:
  7 $$ IMF_{i + 1} = \frac{1}{I}\sum\limits_{i = 1}^{I} {E\left[ {r_{i} (t) + n_{i} (t)} \right]} $$
5. Next, steps (3) and (4) are repeated until the residual component signal cannot be further decomposed, and finally, the *i*-th modal function component is obtained. At this time, the original signal $s(t)$ is described as:
  8 $$ s(t) = \sum\limits_{i = 1}^{I} {IMF_{i} } + r_{I} (t) $$
  where $r_{I} (t)$ is the final residual component.

### ICA algorithm

1. ICA separation model.

ICA is a popular blind source separation approach, which can estimate useful source signals by observing signals with little prior information. The specific model is:

Assume that the observed m-dimensional signal $X{ = }\left[ {x_{1} ,x_{2} ,...,x_{m} } \right]^{{\text{T}}}$ is obtained by linear aliasing of *n* independent signals $s = \left[ {s_{1} ,s_{2} ,...,s_{n} } \right]^{{\text{T}}}$ through an unknown system:

9 $$ x_{i} = \sum\limits_{j = 1}^{n} {a_{ij} } s_{j} {\kern 1pt} {\kern 1pt} {\kern 1pt} {\kern 1pt} {\kern 1pt} {\kern 1pt} {\kern 1pt} {\kern 1pt} {\kern 1pt} {\kern 1pt} {\kern 1pt} {\kern 1pt} i = 1,...,m $$

Equation (9) can be expressed with the following matrix form:

10 $$ X = As $$

where *X* describes the observation matrix, $s$ indicates the signal source matrix, and $A$ represents a $M*N$ mixing matrix composed of unknown mixing coefficients $a_{ij}$.

The ICA algorithm aims to obtain a separation matrix $W$ from the observation matrix $X$ so that $y_{i} (t)$ and $s_{i} (t)$ are infinitely close and make any two output signals $y_{i}$ and $y_{j}$ uncorrelated; that is, to obtain a linear transformation of $y_{t}$ through the matrix $W$, described with the following formula:

11 $$ Y = WX $$

where $W = \left[ {W_{1} ,W_{2} ,...,W_{M} } \right]$ is the unmixing matrix. It is necessary to optimize each weight $W_{i}$ in matrix W through processing such as averaging, initializing, and normalizing the initial signal, and further obtain the estimated value of $Y_{i}$ separated from the signal, as shown in Eq. (12):

12 $$ y_{i} (t) = \sum\limits_{j = 1}^{M} {A_{j} } e^{{( - \alpha_{j} + 2\pi jf_{j} )t}} $$

where $A_{j}$ describes the amplitude, $\alpha_{j}$ is the attenuation factor, and $f_{j}$ is the oscillation frequency.

3. Fast ICA algorithm principle.

This article establishes the Fast ICA algorithm via negative entropy maximization and the Newton iteration method proposed by Hyvarinen to make the ICA algorithm quicker and more convenient. The observed signals in the Fast ICA algorithm should correlate. Therefore, it is first necessary to employ whitening processing to perform correlation processing on the original data to eliminate the correlation between the data to generate independent signals. At the same time, whitening processing can eliminate some Gaussian white noise in the original signal, reducing interference with the data. Next, principal cost analysis (PCA) analysis should be performed to reduce the dimensions of the whitened data and make the dimensions of each data equal, thereby reducing the complexity of the ICA problem. The Fast ICA algorithm includes the following steps:

1. Centralize the observation data $X$ to make its average value zero;
2. Whiten the decentralized data to obtain more standardized data $z$;
3. Choose $m$ independent costs to be estimated, and adjust the corresponding convergence threshold $\varepsilon$;
4. Initialize the data so all $w_{i} (i = 1,2,...,m)$ in the separation matrix $W = \left[ {W_{1} ,W_{2} ,...,W_{M} } \right]$ have a unit norm.
5. Iterate the Newton iteration method $w_{i} \leftarrow E\left\{ {zg(w_{i}^{T} z)} \right\} - E\left\{ {g^{\prime}(w_{i}^{T} z)} \right\}w_{i}$, where $g(y) = \tanh (y)$, simultaneously on each *w*_i_.
6. Orthogonalize the matrix *W*.
7. Verify the convergence of matrix W. If there is no convergence, go to step (5).
8. Separate independent signals from the mixed signal.

### Permutation entropy threshold principle

CEEMDAN-ICA algorithm is utilized to decompose and transform the blasting signal. Due to the noise complexity in the blasting signal, it is necessary to set the identified high-frequency noise to zero and smooth the low-frequency noise. In information theory, entropy is often utilized to deal with information measurement while playing a quantitative role in information. Compared to information entropy and approximate entropy, permutation entropy (PE) can reasonably measure the complexity of sequences under different noise conditions. Therefore, this paper distinguishes noise signals from blasting signals based on permutation entropy. The specific algorithm is^22^:

For any time series $\left\{ {X(i),i = 1,2, \ldots ,d} \right\}$, the phase space reconstruction is accomplished to attain the matrix $M$:

13 $$ M = \left[ {\begin{array}{*{20}c} {x(1)} & {x(1 + \tau )} & \cdots & {x\left[ {1 + \left( {d - 1} \right)\tau } \right]} \\ {x(2)} & {x(2 + \tau )} & \cdots & {x\left[ {2 + \left( {d - 1} \right)\tau } \right]} \\ {x(3)} & {x(3 + \tau )} & \cdots & {x\left[ {3 + \left( {d - 1} \right)\tau } \right]} \\ \vdots & \vdots & \ddots & \vdots \\ {x(i)} & {x(i + \tau )} & \cdots & {x\left[ {i + \left( {d - 1} \right)\tau } \right]} \\ \end{array} } \right] $$

where $\tau$ describes the delay time, $d$ is the embedding function, $n$ indicates the number of reconstructed components, and $i = n - (d - 1)\tau$.

There are $d$ reconstructed components in $M$, which are rearranged in ascending order, and the column index of all components in the obtained vector is arranged according to $\left\{ {j_{1} ,j_{2} , \ldots ,j_{d} } \right\}$; that is:

14 $$ x\left[ {i + (d_{1} - 1)\tau } \right] \le \cdots \le x\left[ {i + (d_{j} - 1)\tau } \right] $$

Each row of matrix $M$ can get the following set of symbol sequences:

15 $$ S(l) = \left\{ {j_{1} ,j_{2} , \ldots ,j_{d} } \right\} $$

where $l = 1,2, \ldots ,d$, and $d \le m!$. $m$ is a different symbol sequence $\left\{ {j_{1} ,j_{2} , \ldots ,j_{d} } \right\}$ of phase space mapping, while there are $m!$ kinds in total.

Next, the probability $\left\{ {P_{1} ,P_{2} , \ldots ,P_{d} } \right\}$ of the occurrence of the employed symbol sequence is computed, which can be described with the following Shannon entropy form:

16 $$ H_{p} (d) = - \sum\limits_{j = 1}^{n} {P_{j} \ln (P_{j} )} $$

The magnitude of the entropy value of $H_{p}$ describes the complexity of the time series $\left\{ {X(i),i = 1,2, \ldots ,d} \right\}$. The more complex the time series, the greater the entropy.

### The combined denoising algorithm (CEEMDAN-ICA)

**Figure 1:**
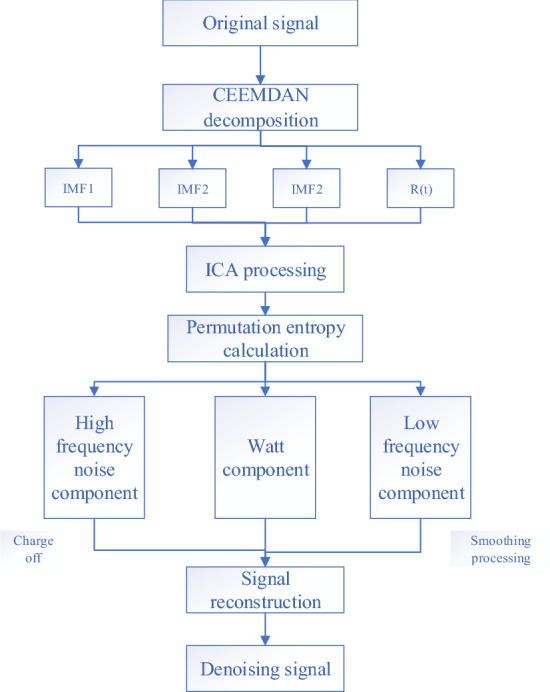
CEEMDAN-ICA algorithm flow chart.

Figure 1 shows the CEEMDAN-ICA joint algorithm’s noise reduction flow chart. As presented in Fig. 1, the blasting vibration signal is first decomposed through the CEEMDAN algorithm, obtaining multiple intrinsic mode components and performing preliminary noise reduction analysis. Then, the ICA algorithm is utilized to further denoise and estimate the original signal. Finally, the permutation entropy algorithm is adopted to calculate the entropy values of different components obtained by the ICA algorithm, and signal reconstruction is performed according to the entropy results.

## Simulation signal simulation verification

### Simulation signal denoising

Simulation signals are employed to evaluate the noise reduction ability of the CEEMDAN- ICA algorithm. Existing research has found that adding Gaussian white noise to an appropriate sine cosine function can simulate actual blasting vibration signals. Therefore, the following simulation signals are utilized to verify the correctness of the above methods:

17 $$ \begin{gathered} x_{1} (t) = 0.8\cos (1.2\pi \times 50t) \hfill \\ x_{2} (t) = 0.3\sin (2\pi \times 50t)\left[ {1 + 2.5 \times \sin (0.5\pi \times 40t)} \right] \hfill \\ x_{3} (t) = 0.15e^{ - 15t} \sin (4\pi \times 50t) \hfill \\ w(t) = x_{1} (t) + x_{2} (t) + x_{3} (t) + n(t) \hfill \\ \end{gathered} $$

where $n(t)$ escribes the Gaussian white noise, and $t \in \left[ {0,0.001,1} \right]$ describes the time steps.

**Figure 2:**
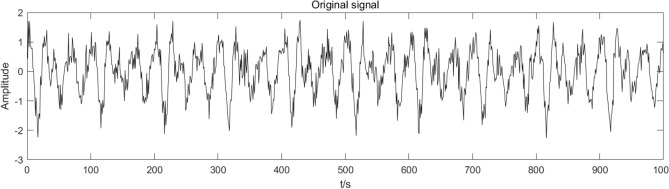
Simulation signal waveform.

The CEEMDAN-ICA algorithm simulates and analyzes the signals in (17). First, the simulated signal’s waveform diagram is obtained, as presented in Fig. 2.

**Figure 3:**
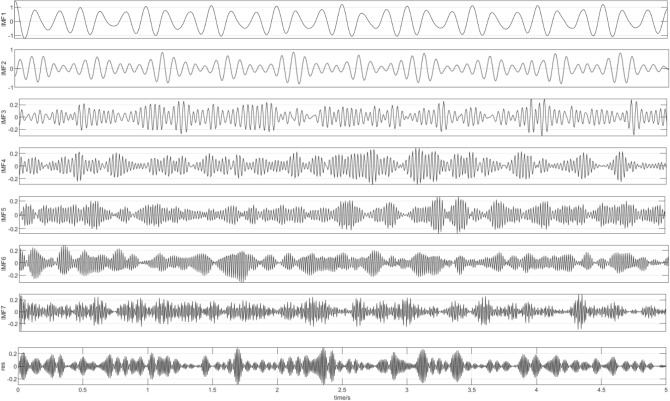
IMF component.

Next, the signal is decomposed by CEEMDAN, 200 groups of noise signals are added to the signal adaptively, and IMF and residual components are obtained under seven groups of different vibration frequencies, as shown in Fig. 3.

**Figure 4:**
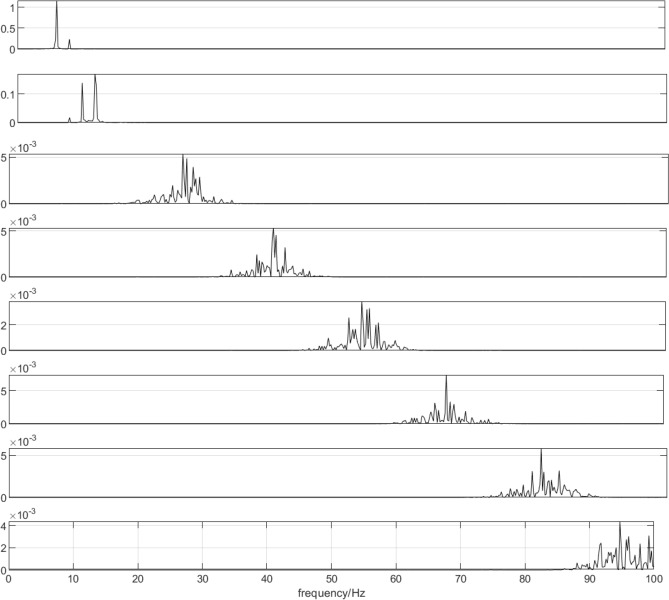
The power spectrum image corresponding to IMF components.

Figure 4 shows the corresponding power spectrum image of each component.

**Figure 5:**
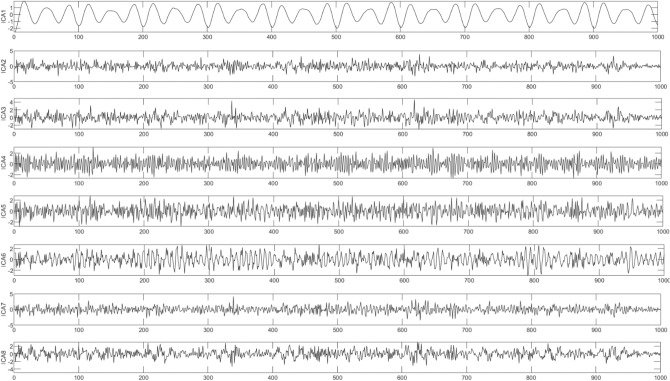
ICA component.

As shown in Fig. 3, the waveform obtained by each decomposition shows that with the continuous progress of time, the signal shows a certain regularity in the whole section, and the increase and decrease of amplitude show a similar symmetric distribution. Therefore, it is proved that the IMF component obtained by CEEMDAN decomposition has a good frequency band separation effect and can effectively avoid the mode aliasing problem. Given the corresponding power spectrum image in Fig. 4, it can be observed that the frequency is continuously increasing, indicating that the decomposition is performed according to a specific power during decomposition. Comparing the IMF component with the original signal indicates a poor correlation between IMF1, IMF2, IMF6, and IMF7 and the original signal and shows that the above four components are noise components. Next, the ICA algorithm is utilized to denoise and estimate the original signal to obtain eight ICA component images, as shown in Fig. 5.

**Figure 6:**
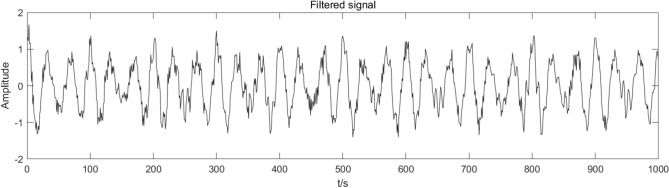
Reconstructed signal.

After calculating the permutation entropy (PE) algorithm, the entropy values of the eight ICA components are obtained as 1.7178, 3.1659, 1.7904, 1.7870, 3.1717, 1.7403, 1.7910, and 3.1392. According to the PE definition, the larger the entropy value, the more complex the time series contained in the signal and the more complex the information contained in the corresponding signal. Therefore, it is necessary to focus on reconstructing complex signals when reconstructing its signal. At the same time, the permutation entropy can further remove the high-noise components during reconstruction and smooth the low-noise components. Figure 6 presents the reconstructed blasting signal.

Comparing the reconstructed signal in Fig. 6 with the simulated signal in Fig. 2 indicates the consistency of the two signals’ waveforms. Given the simulated signal without adding noise signals, the reconstructed signal cannot completely remove the noise component. However, compared to adding noise to the signal, the reconstructed signal denoised by the CEEMDAN-ICA algorithm can effectively eliminate noise components and better restore the original signal.

### Noise reduction effect analysis

In order to evaluate the relationship between the denoised and original signals, SNR and correlation coefficient (COEFE) are defined to compare the denoising effects of several commonly used algorithms. The higher the SNR, the better the denoising impact. The closer the COEFE to 1, the better the correlation between the denoised and original signals.

18 $$ SNR = 10\lg \left\{ {\frac{{\sum\limits_{n = 1}^{N} {x(n)^{2} } }}{{\sum\limits_{n = 1}^{N} {\left[ {x(n) - \hat{x}(n)} \right]^{2} } }}} \right\} $$

19 $$ COEFE = \frac{{N\sum\limits_{n = 1}^{N} {x(n)\hat{x}(n) - \sum\limits_{n = 1}^{N} {x(n)\hat{x}(n)} } }}{{\sqrt {\left[ {N\sum\limits_{n = 1}^{N} {x(n) - \sum\limits_{n = 1}^{N} {\hat{x}(n)^{2} } } } \right]\left[ {N\sum\limits_{n = 1}^{N} {\hat{x}(n) - \sum\limits_{n = 1}^{N} {x(n)^{2} } } } \right]} }} $$

where $x(n)$ and $\hat{x}(n)$ describe the original and reconstructed signals, respectively, and *N* indicates the number of signal sampling points chosen as 1000.

**Figure 7:**
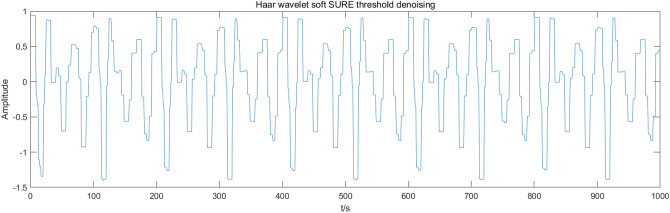
Soft threshold wavelet denoising.

**Figure 8:**
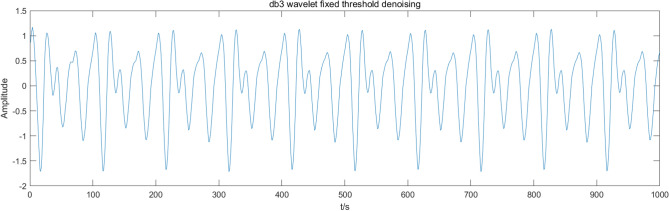
Fixed threshold wavelet denoising.

**Figure 9:**
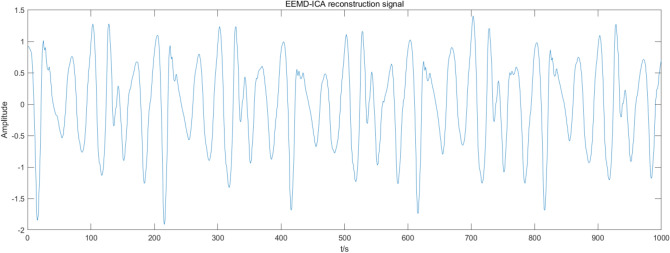
EMD-ICA denoising.

**Figure 10:**
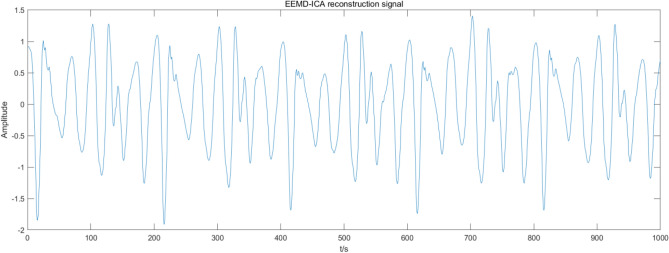
EEMD-ICA denoising.

This paper compares the soft threshold wavelet algorithm, fixed threshold wavelet algorithm, EMD-ICA algorithm, and EEMD-ICA algorithm with the CEEMDAN-ICA algorithm. Figures 7, 8, 9, 10 present the simulation signal processing results.

Figures 7, 8, 9, 10 present the filtering results obtained by applying different algorithms to denoise the simulated signals. The waveform obtained by soft threshold wavelet denoising significantly differs from the original waveform. It shows that more useful information is removed in the denoising process, and the waveform obtained by the fixed threshold wavelet, EMD-ICA, and EEMD-ICA algorithms is similar to the original signal. Then, the SNR and COEFE of the five methods are calculated to compare their advantages and disadvantages. The calculation results are presented in Table 1.

**Table 1:** SNR and COEFE of different algorithms.

Denoising method	*SNR*	*COEFE*
CEEMDAN-ICA	11.2507	0.9757
Soft threshold wavelet	3.2519	0.3481
Fixed threshold wavelet	5.4267	0.7616
EMD-ICA	6.4987	0.8858
EEMD-ICA	6.6713	0.8809

The above results indicate that the CEEMDAN-ICA algorithm results are superior to wavelet, EMD-ICA, and EEMD-ICA algorithms. From the waveform graph, the results obtained by the wavelet algorithm significantly differ from the waveform of the original signal, causing distortion. Although the results obtained by EMD-ICA and EEMD-ICA algorithms are compatible with the original signal in the waveform, many signals are eliminated in the denoising process, affecting the subsequent analysis. Therefore, the CEEMDAN-ICA algorithm has apparent advantages for blasting signals processing.

## Analysis of the measured signal

The CEEMDAN-ICA algorithm decomposes the signal using the actual blasting signal of a tunnel as the analysis object. As presented in Fig. 1, the on-site construction environment and other factors influence the collected blasting signal, the signal has apparent trend items, and its waveform deviates from the baseline center and is distorted. Therefore, preprocessing is required to avoid the adverse effects on subsequent time–frequency analysis of the signal.

**Figure 11:**
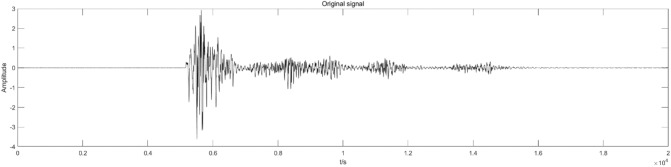
Original signal.

**Figure 12:**
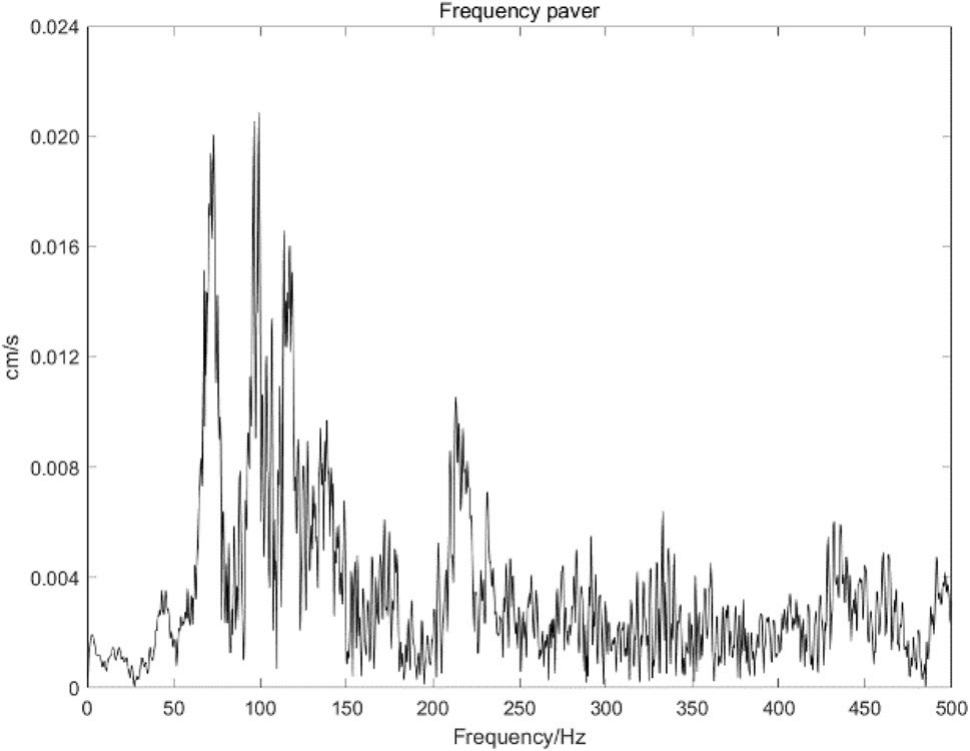
Original signal frequency diagram.

The L20 blasting vibration meter produced by Chengdu Jiaobo Technology Co., Ltd. was utilized to acquire blasting signals. At the same time, preliminary analysis of the collected vibration data was performed using the supporting BVA-L20 intelligent analysis software. The waveform obtained from the acquisition and its corresponding frequency chart are presented in Figs. 11 and 12, respectively.

**Figure 13:**
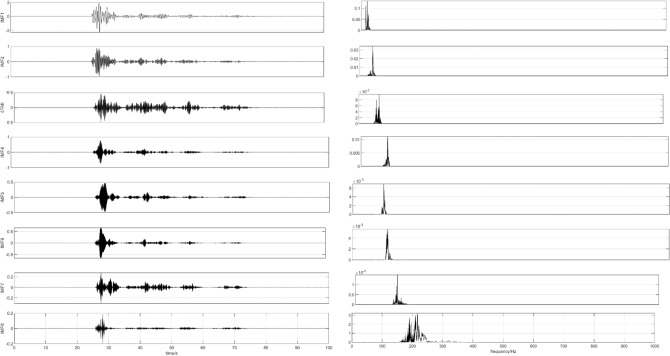
IMF component and power spectrum.

The original signal indicates that the peak value of blasting vibration varies significantly and has noticeable decay and irregularity as it changes over time. According to the existing research, the distribution range of blasting vibration is mainly 50–300 Hz, with a large span. However, its effective frequency is in the range of 30–200 Hz for blasting vibration. Blasting vibration signals should be denoised to analyze vibration signals accurately. Repeated experimental comparisons indicate that adding 200 sets of white noise to the signal can effectively suppress modal aliasing. CEEMDAN decomposition of the original signal is accomplished to obtain seven sets of IMF and residual components. At the same time, Fig. 13 presents the corresponding power spectrum images. As presented in Fig. 13, the power spectrum images corresponding to the decomposed IMF components are arranged according to the frequency from small to large.

**Figure 14:**
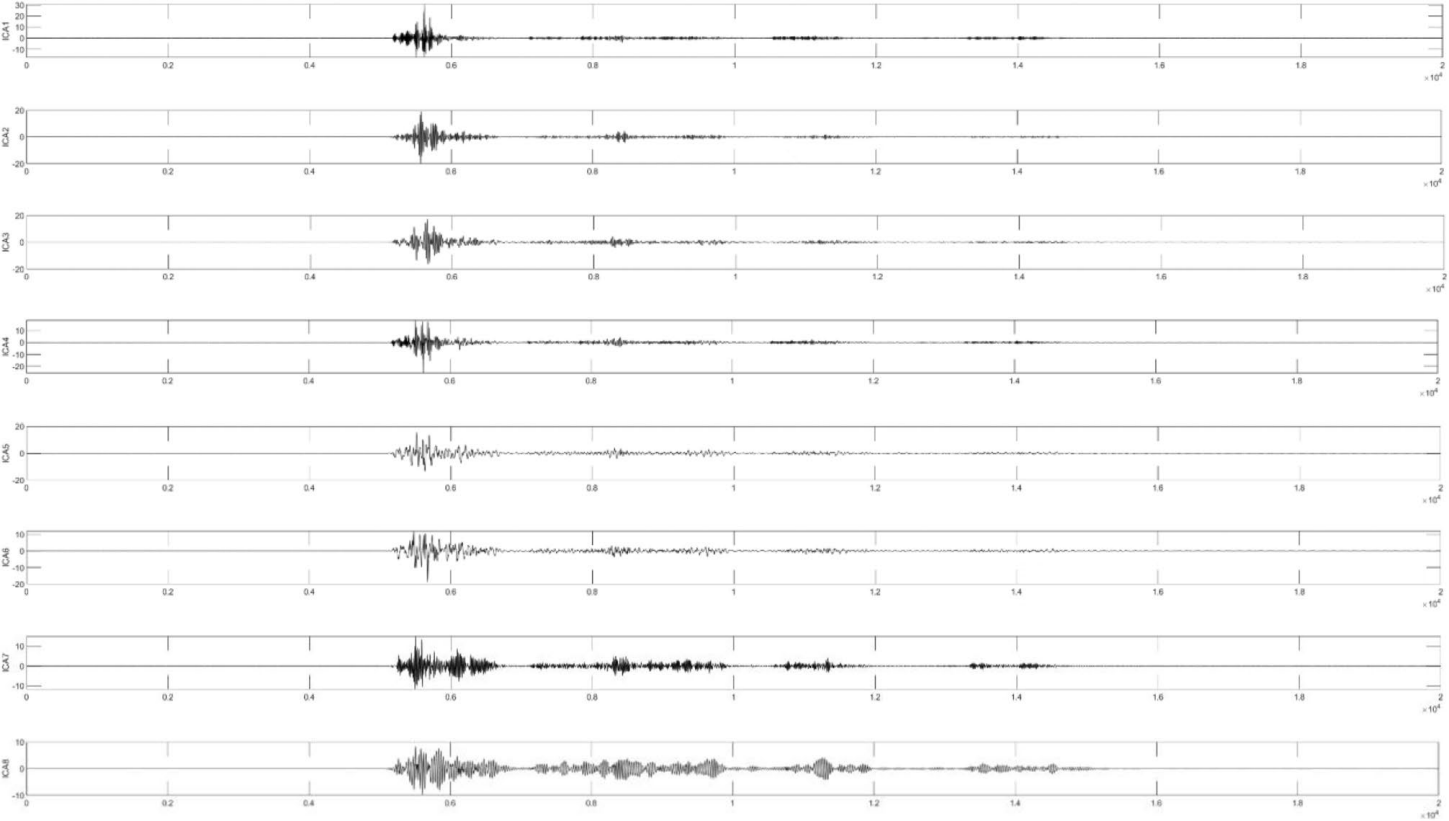
ICA component.

Next, ICA decomposition is performed on the IMF component to obtain eight sets of ICA components. The signal is further denoised during this process, and its ICA components are shown in Fig. 14.

**Figure 15:**
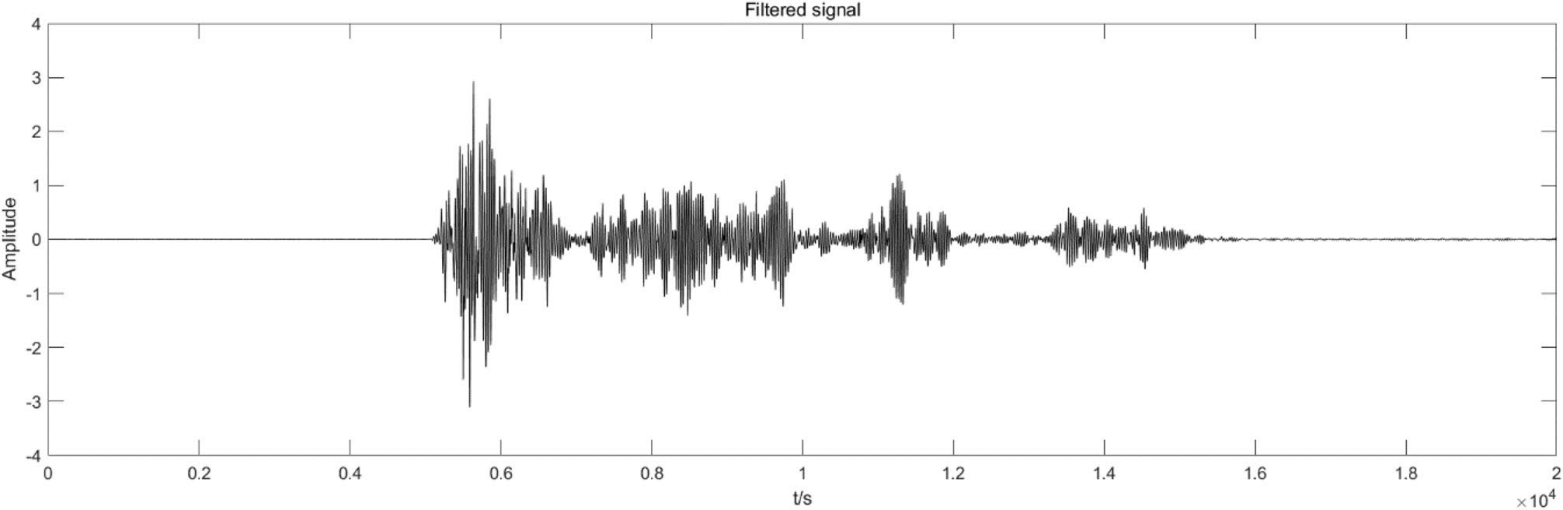
Blasting signal reconstruction.

Finally, the signal is reconstructed according to the entropy value calculated by the arrangement entropy to obtain the reconstructed blasting vibration signal image, as presented in Fig. 15.

**Figure 16:**
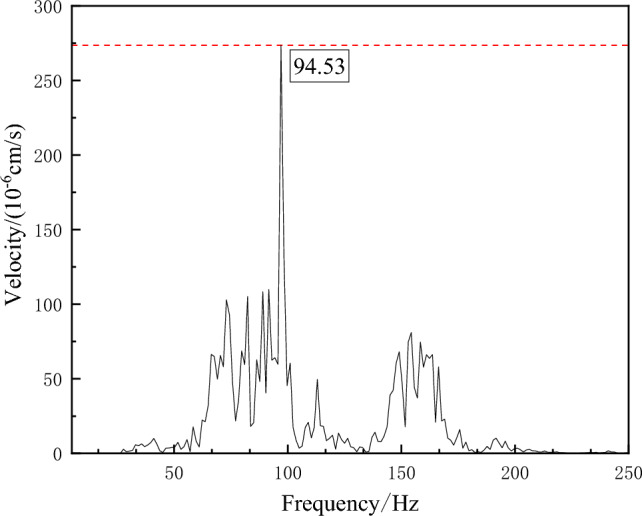
The power spectrum image of the reconstructed signal.

**Figure 17:**
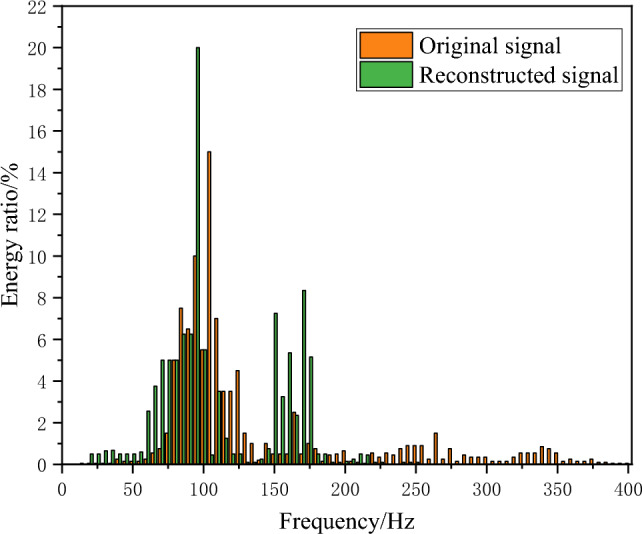
The energy ratio of the original and reconstructed signals.

From the waveform perspective, although the reconstructed blast signal has the same waveform as the original signal, it is smoother and has fewer burr signals than the original signal. In order to further accurately analyze the difference between the reconstructed and original signals, the reconstructed signal’s frequency spectrum image is obtained through the fast Fourier transform (FFT). The results are shown in Fig. 16, and the energy ratios under different frequency bands are extracted, as shown in Fig. 17.

After denoising, the blasting signal’s dominant frequency is mainly between 50 and 115 Hz, while the original blasting signal’s dominant frequency is between 70 and 250 Hz. Combining the percentages of different frequency bands indicates that the low-frequency band energy in the reconstructed blasting signal significantly increases compared with the original signal. In contrast, the percentage of energy in the high-frequency component exhibits a significant decrease. For existing tunnel lining structures near newly built tunnels, blasting frequencies concentrated between 50 and 115 Hz can ensure the structure’s safety.

## Conclusion

The current paper presents a joint denoising algorithm based on the CEEMDAN and ICA algorithms to remove noise from blasting vibration signals. First, the algorithm’s feasibility was verified through simulation experiments and compared with existing algorithms. Finally, the actual signal was analyzed and processed. The following conclusions can be drawn:

1. The CEEMDAN-ICA joint algorithm can efficiently eliminate the noise in the blasting signal and retain the original signal’s morphological features. The filtered signal’s waveform is compatible with the original one.
2. The CEEMDAN-ICA algorithm is compared with EMD-ICA, EEMD-ICA, and wavelet algorithms. The results indicate the superiority of the results of the CEEMDAN-ICA algorithm to other similar algorithms.
3. The processing results of the measured blasting signal indicate that the CEEMDAN-ICA algorithm can effectively remove the high-frequency signal. Besides, the denoised signal can reflect the natural characteristics of blasting vibration, which provides convenience for further blasting vibration hazard analysis.

## Data Availability

The datasets used and/or analyzed during the current study are available from the corresponding author on reasonable request.
